# A novel screen for genes associated with pheromone-induced sterility

**DOI:** 10.1038/srep36041

**Published:** 2016-10-27

**Authors:** Alison L. Camiletti, Anthony Percival-Smith, Justin R. Croft, Graham J. Thompson

**Affiliations:** 1 Biology Department, Western University, 1151 Richmond Street, London, Ontario, N6A 5B7 Canada; 2 Department of Ecology and Evolution, Biophore, UNIL-Sorge, University of Lausanne, 1015 Lausanne, Switzerland

## Abstract

For honey bee and other social insect colonies the ‘queen substance’ regulates colony reproduction rendering workers functionally sterile. The evolution of worker reproductive altruism is explained by inclusive fitness theory, but little is known of the genes involved or how they regulate the phenotypic expression of altruism. We previously showed that application of honeybee queen pheromone to virgin fruit flies suppresses fecundity. Here we exploit this finding to identify genes associated with the perception of an ovary-inhibiting social pheromone. Mutational and RNAi approaches in *Drosophila* reveal that the olfactory co-factor *Orco* together with receptors *Or49b*, *Or56a* and *Or98a* are potentially involved in the perception of queen pheromone and the suppression of fecundity. One of these, *Or98a*, is known to mediate female fly mating behaviour, and its predicted ligand is structurally similar to a methyl component of the queen pheromone. Our novel approach to finding genes associated with pheromone-induced sterility implies conserved reproductive regulation between social and pre-social orders, and further helps to identify candidate orthologues from the pheromone-responsive pathway that may regulate honeybee worker sterility.

The evolution of altruism has long intrigued biologists interested in the origins of behavioural diversity. Reproductive altruism of the type typical of sterile worker and defensive castes of the social insects is obviously costly to the self-less individual, but nonetheless is predicted to evolve via indirect fitness effects^1^. Despite this central prediction from inclusive fitness theory we do not yet have a good understanding of which genes are under indirect selection or that are otherwise involved in mediating the expression of altruistic behaviour^2^,^3^,^4^. The European honey bee *Apis mellifera* is a post-genomic eusocial model^5^,^6^ that has been used to generate lists of genes implicated in worker sterility, mostly from microarray screens for genes responsive to ovary-inhibiting queen mandibular pheromone^7^,^8^,^9^. QMP is an honest signal of queen fecundity to which workers respond by de-activating their ovaries and otherwise adopting alloparental roles within their kin-based colonies^10^,^11^. The progress from array and other honeybee genomic studies^12^ has only begun to identify the most up-stream pheromone-responsive genetic elements through which workers regulate their ovaries in response to QMP. We have recently discovered however that application of QMP to virgin fruit flies suppresses fecundity in a manner comparable to its normal effect on honeybees. Treated flies tend to have smaller ovaries that contain fewer mature eggs^13^,^14^. This worker-like response from *Drosophila melanogaster* (Diptera) to a eusocial honeybee (Hymenoptera) pheromone implies conserved reproductive regulation between social and pre-social orders and introduces *Drosophila* as a proxy model for the action of sterility-inducing pheromones^15^.

In this study we use a *Drosophila* model to screen for loci involved in the olfactory response to queen mandibular pheromone. First, we use a bioassay to quantify the extent to which the fly’s response to QMP is strictly olfactory, as opposed to gustatory or tactile. Second, we screen the near-full complement of *Drosophila melanogaster* olfactory receptors (ORs) via RNAi-mediated knock-downs. Individual ORs that block the fly’s conspicuous worker-like response to QMP represent functional candidates for the olfactory perception of ovary-inhibiting queen pheromone. Finally, to the extent that receptors identified from the fly are homologous to those from the bee, we use our *Drosophila* model to identify, for the first time, candidates from the pheromone-responsive pathway that may regulate honeybee worker sterility.

## Results and Discussion

**Figure 1:**
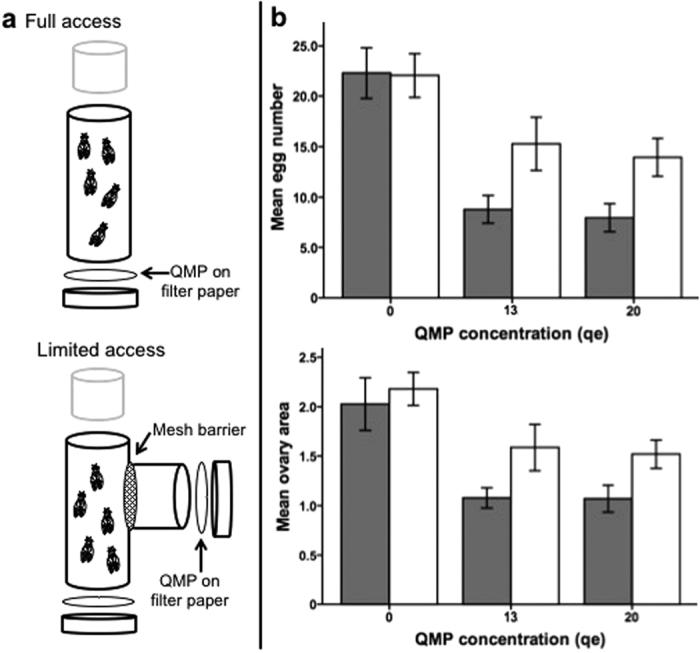
Exposure and response to QMP. (**a**) We used ‘full’ or ‘limited’ access chambers to expose groups (n = 5) of wild type flies to filter paper containing queen mandibular pheromone (QMP) or a no-QMP control. In full access chambers, flies could touch the filter paper; under limited access, they could not. (**b**) Response of wild type flies to QMP under full (gray bars) or limited (white bars) access. Both response variables (egg number, ovary area) decrease by 20 to 64% under QMP treatment, and this response holds in the limited access condition. Error bars indicated 95% confidence intervals.

To determine whether the action of QMP in flies required direct physical contact, we set up two trails. Under “full access” trails we used custom-built chambers that permitted full physical contact with a pheromone-treated filter paper. Under “limited access” trails, by contrast, we fit a screen within chambers that prevented contact with the pheromone (Fig. 1A). Flies exposed to QMP consistently showed a worker-like response. In full access trials females are observed to regularly make contact with the pheromone-treated filter paper and produced smaller ovaries (*F*_2,53_ = 16.74, *P* < 0.001) that contained fewer eggs (*F*_2,67_ = 78.91, *P* < 0.001) than did untreated controls. Even with limited access (separated by ~4 cm) to QMP females yielded smaller ovaries (*F*_2,52_ = 17.19, *P* < 0.001) with fewer eggs (*F*_2,67_ = 37.95, *P* < 0.001) than did non-exposed controls (Fig. 1B). This latter response strongly implicates QMP as a near-distance olfactory cue for suppression of direct fitness.

**Figure 2:**
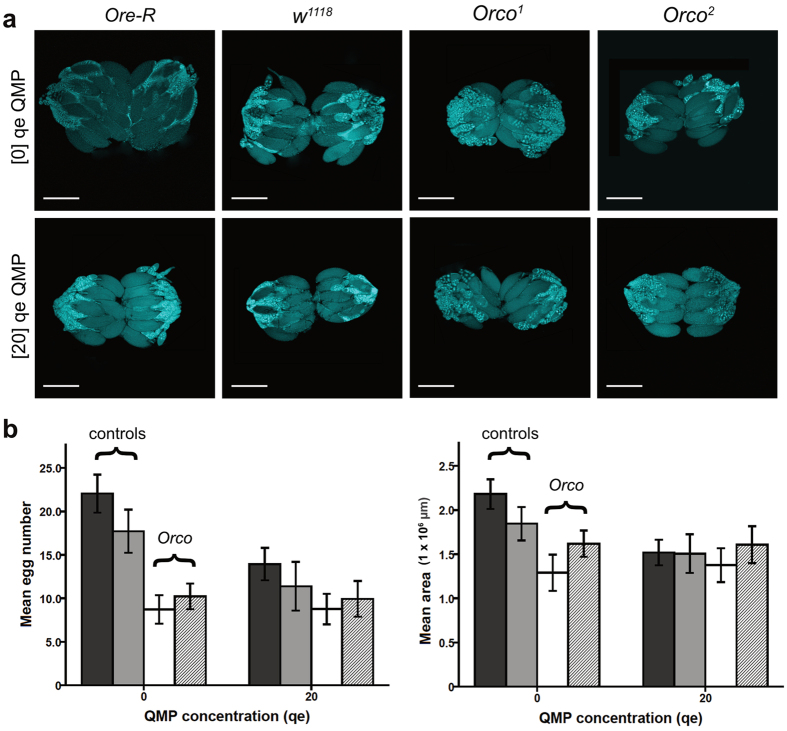
Measuring *Orco* mutant response to QMP. (**a**) Confocal images of *Drosophila* ovaries stained with DAPI. Wild type (*Ore-R*) and background control (*W*^*1118*^) genotypes have relatively large, well-developed ovaries under a zero ‘queen-equivalent’ dose of QMP that regress upon exposure to a [20] qe dose. The ovaries of *Orco* mutants (*Orco*^1^, *Orco*^2^), by contrast, are not affected by QMP. (**b**) Bar graphs summarize how control lines (*Ore*-R, black; *w*^*1118*^ solid grey) respond to QMP while mutant lines (*Orco*^1^ white; *Orco*^2^ striped) do not. This genotype × treatment effect is significant for both measures of fecundity (egg number, ovary area). Scale bar = 200 μm.

To test whether olfaction is required for perception of QMP we compared the ovarian response of small groups (n = 5) of *Ore-R* females against two mutant genotypes deficient for the major olfactory co-factor *Orco* (formerly, *Or83b*). The mutant genotypes are: *w*^*1118*^; *Orco*^1^ and *w*^*1118*^; *Orco*^2^. They are each homozygous for loss-of-function alleles^16^ characterized by a coding region deletion, and both are effective at blocking a wide range of olfactory stimuli^17^. *Orco* females have inherently smaller ovaries^18^ and did not respond to QMP, unlike the background (*w*^*1118*^) or wild types (*Ore-R*) that did contain smaller ovaries (*F*_3,145_ = 7.26, *P* < 0.001) with fewer eggs (*F*_3,144_ = 8.90, *P* < 0.001; Fig. 2). This lack-of-response suggests that *Orco* – a 1-to-1 orthologue with the bee’s *AmOr2*^19^, which itself is up-regulated in sterile workers^7^ – is essential for the perception of QMP and its downstream effect on ovaries.

**Figure 3:**
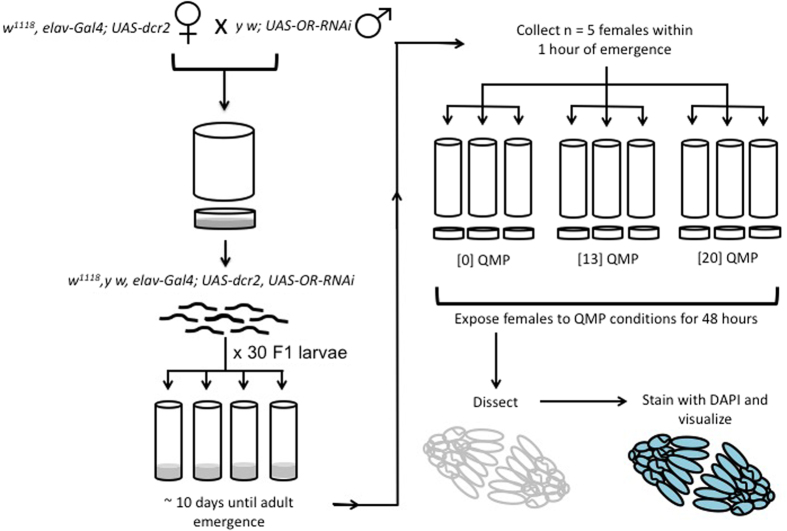
Exposure of RNAi flies to queen bee pheromone. We crossed *elav-GAL4; UAS-dcr2* females to males with specific *UAS-OR-RNAi* genotypes, corresponding to the n = 48 OR knockdowns available for *Drosophila* (from Vienna Drosophila RNAi Stock Center). We collected groups of n = 30 F1 larvae and reared them for ~10 days to maturity. We then exposed small groups (n = 5) of mature same-aged (within 1 hr) females to the QMP treatment. Flies received either no-QMP, or a low [13.3 queen-equivalents] or high [20 queen-equivalents] dose. Finally, after 48 hrs we dissected complete sets of ovaries, stained them with DAPI, and scored them against an established scale^38^ for assessing reproductive readiness, and did so via digitized confocal images.

To identify olfactory receptors (ORs) that “tune” *Orco* to the specific perception of QMP we systematically knocked down individual ORs via Gal4-driven RNAi insertions^20^ available from Vienna Drosophila RNAi Center. We crossed *UAS-RNAi* males of either P-element RNAi (“GD Library”) or phiC31 (“KK Library”) genetic background with virgin *w*^*1118*^, *elav-Gal4; UAS-dcr2* females to generate F1s that express OR-specific knockdowns (Fig. 3). We proceeded with lines only for which the background had a minimal effect on ovary phenotype. To select for this, we compared ovary scores for each RNAi line to background control F1s produced from crossing *elav-Gal4; UAS-dcr2* females to GD or KK males (with no QMP). From each pairwise comparison (Supplementary Table 1), we considered only those knockdown lines for which the standardized difference in mean ovary scores was ‘small’ - *i.e.*, Hedge’s *g* less than 0.5 (Supplementary Table 2). The majority of lines screened met this criterion (34 of 45 lines for egg number; 26 of 45 lines for ovary area) and were deemed suitable for testing the knockdown effect of specific ORs against QMP. We again measured this effect using Hedge’s *g*, except in this case by simply comparing QMP treated vs. untreated flies.

**Figure 4:**
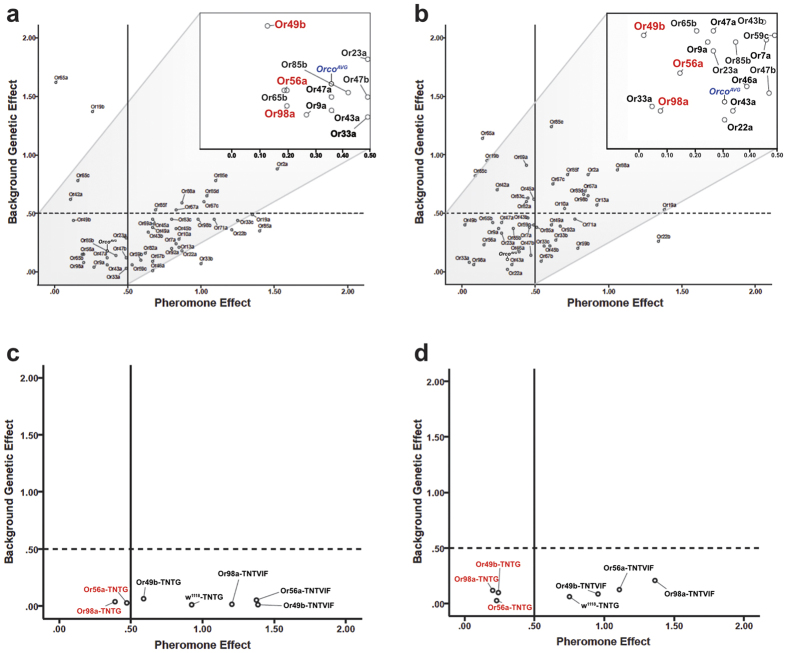
RNAi screen for olfactory receptors responsive to queen pheromone. For each *Drosophila* olfactory receptor RNAi knockdown line (n = 48) we show the statistical effect size (Hedge’s *g*) of pheromone treatment (*x*-axis) and genetic background (*y*-axis) on egg number (**a**) and ovary area (**b**). RNAi lines that map to the lower left quadrant are hardly responsive to QMP (*g* < 0.5 on *x*) and have low genetic backgrounds (*g* < 0.5 on *y*). Three ORs in red have particularly strong knockdown effects (*g* < 0.2 on *x*) and are retrieved from both assays independently. We superimpose the *Orco* mutant effect for comparison. To validate the RNAi-implied function of the three highlighted receptors we used promoters for each of Or49b, Or56a and Or98a fused to *GAL4* to drive expression of a tetanus toxin transgene (i.e., -TNTG) alongside an inactive toxin control (i.e., -TNTVIF). We find that this targeted disruption of neural activity effectively mimics the RNAi-knockdown effect for two of the three receptors tested: Or56a and Or98a are required to mediate the full pheromone response on egg number (**c**) and ovary area (**d**), while Or49b is seemingly required only for ovary area *g* < 0.5, (**d)** only. The wild type *w*^*1118*^-TNTG and inactive toxin lines (Or56a-TNTVIF, etc) show the typical very large effect of queen pheromone on fly egg number and ovary area.

**Figure 5:**
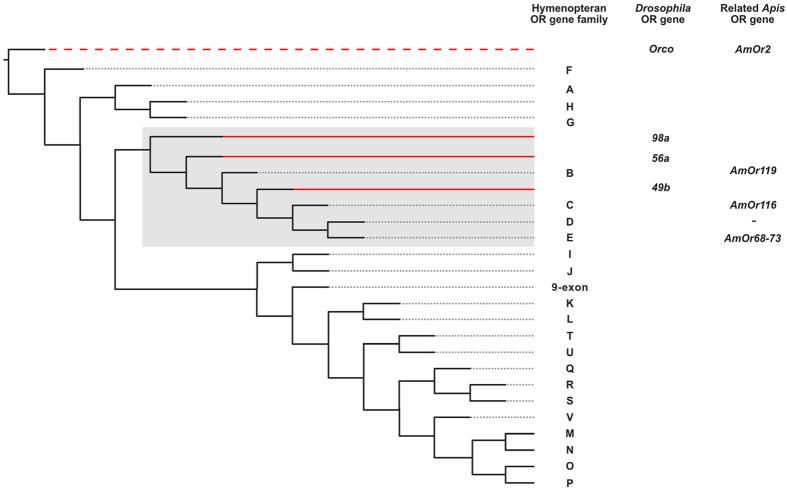
Most highly related *Apis* olfactory receptors to the candidate *Drosophila* ORs identified in our screen. Genealogical relationship between Hymenopteran olfactory receptor families (A-P, 9-exon, *Orco*) and the *Drosophila* olfactory receptors identified from the present screen (n = 4, incl. *Orco*). Red shows *Drosophila* genes (*Or49b*, *Or56a*, *Or98a*) embedded within the Hymenopteran genealogy, with dashes indicating 1-to-1 orthology between *Orco* and *AmOr2*. These relationships are re-drawn from Zhou *et al*^21^ (Supplementary Figure 3 therein) and are here used to identify the most-closely related *Apis* genes (n = 9, incl. *AmOr2*).

For egg number, n = 23 lines had no appreciable knockdown effect on the perception of QMP – that is, females continued to show a worker-like response. The remaining lines did, however, show a strong knockdown effect as evidenced by lack-of-response to QMP (Fig. 4). For ovary area, n = 10 RNAi lines had no appreciable knockdown effect and thus continued to show the worker-like response. The remaining lines did, however, show a strong knockdown effect on the expected response to QMP. In total 16 unique ORs are identified from the two ovary-related assays, with some overlap. We noticed that three of the olfactory receptors – named *Or49b*, *Or56a* and *Or98a* – are retrieved from both assays independently, and have particularly strong knockdown effects as evidenced by lack-of-response to pheromone (*g* < 0.2; Fig. 4A,B). These three *Drosophila* receptors are therefore strongly and consistently associated with pheromone-induced ‘sterility’, and cluster on a genealogy^21^ with Hymenopteran OR gene subfamilies “B”, “C”, “D” and “E” (so named in ref. 21). Collectively, these gene subfamilies each potentially represent one OR gene copy in the common ancestor of Hymenoptera and contain a mere eight (of ~170)^19^ extent *Apis mellifera* olfactory receptor orthologues, which are: *AmOr116*, *AmOr119* and *AmOr68*-*AmOr73* (Fig. 5). Thus in addition to *AmOr2* identified above, our screen implicates a clear set of n = 8 honeybee genes in the QMP-responsive pathway that may regulate *Apis mellifera* worker sterility. It is not yet feasible to knockdown individual olfactory receptor genes in the sensory tissues of the bee itself. This technology is being developed^22^,^23^ and we predict individual or collective knockdown of these eight bee receptors in a native context will inhibit the worker’s altruistic response to queen pheromone.

We independently tested the RNAi-derived results in the fly model using promoters for each of Or49b, Or56a and Or98a receptors fused to *GAL4* to drive expression of the tetanus toxin, and scored the female’s reproductive response to pheromone. As predicted, the receptor-specific disruption of synaptic function dampens the response to queen pheromone, producing females with enlarged ovaries (*F*_1_ = 53.63, *P* < 0.001) that contain more eggs (*F*_1_ = 87.86, *P* < 0.001) than do non-toxic transgene controls (Fig. 4C,D). Why olfactory receptive flies respond to bee pheromone is an open question^13^,^14^, with one possibility being that social insect queen pheromones act on conserved regulatory pathways that were already present in solitary ancestors^24^. Regardless, our tetanus toxin-based validation suggests that the original RNAi screen did capture a functional subset of receptors with a capacity to perceive the ovary-inhibiting pheromone. Our receptor- and neuron-specific rescue is simply not expected under a non-specific pharmacological response to a pheromone of any type. Further testing of pheromone-treatment specificity is possible – for example, by testing flies for any gains-of-function predicted from honey bee biology upon exposure to QMP (e.g., behavioral attraction to pheromone). *Or49b and Or98a* are broadly tuned receptors that respond to a range of ecologically relevant odors^25^. *Or56a*, by contrast, is apparently very narrowly tuned but its function is nonetheless linked to ovary inhibition^26^, which is clearly relevant to ‘sterility’. An electrophysiology or activity assay^27^ will help clarify if fly olfactory neurons expressing these three receptors are actively and specifically detecting the bee pheromone.

**Figure 6:**
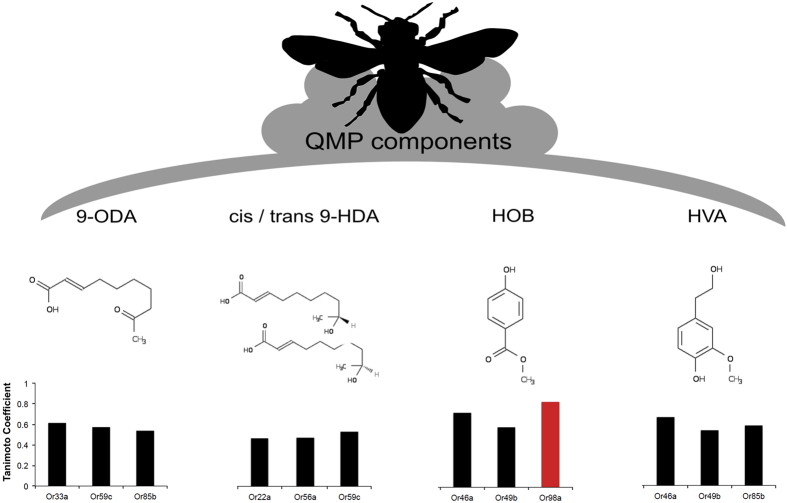
Structural similarity between QMP molecular components and predicted receptor ligands. Queen mandibular pheromone consists of five organic components (9-ODA, cis/trans 9-HDA, HOB and HVA) that show sub-structural similarity to the principle ligands of *Drosophila* olfactory receptors, as measured here using Tanimoto’s coefficient (Supplementary Table 3). The ligand for *Or98a* (in red) has a strong affinity for the HOB component of queen pheromone.

Finally, we used the on-line Database of Odorant Receptors^28^ and the maximum common sub-structure method of Cao *et al*.^29^ to predict the affinity of predicted receptor ligands to any of the five components of QMP (Supplementary Table 3). *Or98a*, identified above, showed the single highest sub-structural similarity score to any component of QMP (to methyl *p*-hydroxybenzoate, HOB; Fig. 6). The conspicuous response from the fly to honeybee pheromone may, therefore, lie in conserved olfactory or other^30^ signaling mechanisms that remain linked to female fecundity and ovary de-activation, as predicted by socio-evolutionary hypotheses^24^,^31^,^32^,^33^. *Or98a* and HOB may indeed be linked to reproduction: the former mediates female fly mating behaviour^34^ and the latter molecule varies in its expression as a function of reproductive caste across *Apis* spp.^35^. Whether HOB or other single components of the queen pheromone blend can singlehandedly induce sterility in flies has not been widely tested^36^, but data emerging from honeybees does suggest that some single-components can suppress worker fertility^24^.

## Conclusions

Our findings advance insect sociobiology in two ways. First, we demonstrate how a novel *Drosophila* model can accelerate discovery of genes relevant to the evolution and expression of socially-mediated reproduction. Second, we implicate *Orco* and its *Or49b*, *Or56a* and *Or98a* receptors as functional orthologues in the pheromone-responsive pathways that may regulate honeybee worker sterility – a pathway of major significance to insect sociobiology.

## Methods

### Fly rearing

We reared all strains of *Drosophila melanogaster* under standard conditions (25 °C, 60% humidity and a 12 h:12 h light: dark cycle) in an insect growth chamber (Caron Inc., Marietta, OH) on a standard cornmeal diet, as described in ref. 14. We synchronized adult emergence by first housing (for 24 hrs) a small reproductive population (n = 30 males and n = 30 females) in collection cages (60 mm; Diamed, Mississauga, Canada) fitted with nutrient (grape juice and agar) plates. We then collected and transferred day-old larvae to fresh food vials (28.5 × 95 mm, VWR International, Radnar, PA) at a density of n = 30 larvae per vial. Finally, we collected same-age (within 1 h) adult virgin females approximately 10 days later, and dissected their ovaries after a further 48 hours.

### Pheromone treatment

First, we diluted a 500 mg stock of synthetic QMP (Contech Ltd, Victoria, Canada) with 100% ethanol into two working concentrations; a relatively low dose of approximately 13 queen equivalent (qe) units^37^ and a higher dose of approximately 20 qe units. We use doses that are nominally high as prepared within the fly food (yeast and sugar) medium, but the amount actually received by flies coming in contact with the medium-soaked filter paper (in groups of five) is presumably much less. We reason that if female flies consume roughly 2 μl of food per day then the oral dose of QMP would be ~1.3 queen equivalents (13 QE in 20 μl @ 2 μl = 1.3 QE), or less via olfaction alone. Therefore, the effective dose is estimated to be within a biologically relevant range that has been shown to effectively suppress fly ovaries in a manner comparable to its normal effect on worker bees^14^. Moreover, the effect of pheromone treatment at these doses does appear to specifically affect fly ovaries, as opposed to other aspects of female reproduction (pupation) or survivorship^14^. Lack-of-response to a control pheromone (7-triclosene), and variable response among reproduction-related genotypes^13^, further suggests that this ovary-specific response is not simply a pharmacological side-effect to a pheromone of any type. Second, we warmed working aliquots to 50 °C in a water bath, and exposed flies to QMP in one of following two ways. Under “full access” we exposed flies to QMP within chambers that permitted full physical contact with the pheromone-treated filter paper. Under “limited access”, by contrast, we exposed flies to QMP within chambers fitted with a screen that prevented physical contact with the pheromone-treated filter paper.

For full access trials we placed n = 5 flies into a 50 ml Falcon tube modified to administer pheromone, as described in ref. 14. Briefly, we cut the bottom tip of the tube to insert a standard fly plug, then custom fit a piece of filter paper (grade 413: VWR International, Radnar, PA) saturated with a yeast and sugar solution (0.1 g yeast, 0.15 g sugar, in 5 ml of 5% ethanol) under the screw cap. To treat flies, we pipetted 20 μl of QMP-EtOH solution, or the equivalent volume of just-EtOH control, onto the paper, and incubated the whole chamber for a period of 48 hrs. For limited access trials, we performed a comparable procedure, except used a mesh barrier to prevent flies from touching the filter paper. Following treatment, we dissected the ovaries of individual flies, and scored the approximate level of activation in two ways; by counting the number of mature eggs^38^ per female (both ovaries) and by estimating the total ovary area, as inferred from on-screen measurements of digitized confocal microscope images.

### Scoring the level of ovary activation

For all assayed flies, we exposed females within chambers for 48 hrs. We then CO_2_ anesthetized them and dissected complete pairs of ovaries from individual females using ultra-fine forceps under an Olympus S7 × 7 stereomicroscope (Olympus, Richmond Hill, Canada) that we fitted with a cold light source (KL300 LED; Leica Microsystems, Wetzlar, Germany). We dissected ovaries in 1X Dulbecco’s phosphate-buffered saline (1 × D-PBS; Invitrogen, Carlsbad, CA), then fixed stained tissue in a 4%-formaldehyde in D-PBS solution for a period of 20 min. We then washed [1 × D-PBS and 0.5% PBT (0.1% Triton X 100 in 1 × D-PBS)] and DAPI-stained (1:2000) ovaries prior to mounting (7% glycerol in D-PBS) and visualized them using a Zeiss LSM 5 Duo Vario confocal microscope (Zeiss, Oberkochen, Germany). Finally, we scored ovaries against two biological criteria that capture QMPs effect on reproductive readiness. First, we counted the number of mature (stage 14)^38^ eggs within each ovary. We then estimated ovary area (μm^2^) from confocal images using the ‘thresholding’ function of Image-Pro Premier 9.1 software (Version 9.1, Media Cybernetics, Bethesda, MD). For this part of the analysis, we excluded any ovaries that were inadvertently damaged during dissection or that otherwise had weak imaging (a minority, ~3–5%).

### Electrochemical disruption of synapse via GAL4-driven tetanus toxin

To validate the RNAi knockdown effects on individual olfactory receptors, we drove a tetanus toxin transgene using promoters specific to receptors *Or49b*, *Or56a* and *Or98a*. These *Or49b*-GAL4, *Or56a*-GAL4, *Or98a*-GAL4 driver lines were obtained from Bloomington Stock Center. The active form of the tetanus toxin transgene (TNTG) inhibits neural function by blocking synaptic transmission^39^. The tissue-specific delivery of this effect into our top three ORs of interest (*See* Fig. 4A,B) provides an independent test of their physiological capacity to mediate the ovary-inhibiting effect of queen pheromone. Specifically, we crossed *w*; *Or-GAL4* males with *w; UAS-TNTG* females to generate F1s that express the OR-specific neural disruption (e.g., *w;* Or98a-GAL4/UAS-TNTG). As a background control, we likewise crossed *w*; *Or-GAL4* males with *w; UAS-TNTVIF* females carrying the inactive form of the toxin, and proceeded with only those lines for which the background had a very minimal effect on ovary phenotype (Hedges *g* less than 0.2). We predict that the synaptic disruption of specific olfactory receptors via tetanus toxin will mimic the original RNAi knockdown effect to render female flies significantly less responsive to ovary-inhibiting pheromone. The rearing, treatment and scoring of fly populations was identical to that for the RNAi experiment (above).

### Calculating statistical effect size

Effect size comparisons were computed using


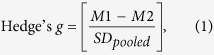


an unbiased version of Cohen’s *d* index of effect size for sample sizes smaller than n = 20 (Hedges, L. V. 1981. Distribution theory for Glass’s estimator of effect size and related estimators. Journal of Educational Statistics, 6, 107–128), whereby *M* is the population mean of each response variable, the pooled standard deviation SD of both samples is


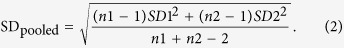


### Stoichiometric analysis of olfactory ligands to pheromone components

Following our screen, we compared the structural similarity of candidate olfactory receptor ligands to the five components^40^ of QMP: 9-ODA (E)-9-oxodec-2-enoic acid), HOB (methyl *p*-hydroxybenzoate), HVA (4-hydroxy-3-methoxyphenylethanol) and *cis* and *trans* 9-HDA (9-hydroxydec-2-enoic acid). First, we identified the dominant ligand for each candidate OR using the on-line Database of Odorant Receptors^28^. In four cases (corresponding to *Or47a*, *Or43a*, *Or43b* and *Or22a*) there was more than one probable ligand (Supplementary Table 3). We then used the maximum common sub-structure (MCS) method of Cao *et al*.^29^ to predict the affinity of ligands to individual components of QMP. For each test, we used the ChemMine application^41^ that generates a Tanimoto^42^ ‘similarity score’ for each pair of compounds (MSC Ts). In this context, a high score (maximum of ‘1’) implies a higher chemical identity between fly ligand and bee pheromone. In total we identified n = 21 ligands corresponding to the 16 unique receptors identified from our RNAi screen. The structural similarity scores between ligand and QMP component ranged from 0.20–0.83, suggesting that sub-structural analysis contains ample variation to predict biological affinity.

## Additional Information

**How to cite this article**: Camiletti, A. L. *et al*. A novel screen for genes associated with pheromone-induced sterility. *Sci. Rep.* **6**, 36041; doi: 10.1038/srep36041 (2016).

**Publisher’s note:** Springer Nature remains neutral with regard to jurisdictional claims in published maps and institutional affiliations.

## Supplementary Material

Supplementary Information
