# Supplementary material for: A novel screen for genes associated with pheromone-induced sterility
Source: Sci Rep. 2016 Oct 27;6:36041. doi: 10.1038/srep36041 (PMC5081541; doi:10.1038/srep36041)
Supplement: Supplementary Information [file srep36041-s1.doc]

**Supplementary Information**

**A novel screen for genes associated with pheromone-induced sterility**

Alison L Camiletti^1^, Anthony Percival-Smith^1^, Justin R Croft^1^ & Graham J Thompson^1, 2^

^1^ Biology Department, Western University, 1151 Richmond Street, London, Ontario, Canada N6A 5B7

^2^ Department of Ecology and Evolution, Biophore, UNIL-Sorge, University of Lausanne, 1015 Lausanne, Switzerland

**Supplementary Table 1. Summary of the olfactory RNAi line genotypes used in the pheromone screen. We show the olfactory receptor common name, the corresponding Flybase Gene ID, the Vienna *Drosophila* Resource Center (VDRC) ID, and the background Library ID from which the transgenic fly was made.**

|  |  | UAS-RNAi Strain (**male**) | | | |
| --- | --- | --- | --- | --- | --- |
|
|  |  | Common Name | Gene ID | VDRC ID | Library ID |
| w^1118^, elav-Gal4; UAS-Dcr2 (**female**) | *crossed to:* | Or85a | CG7454 | 14099 | GD |
|  |  | Or22a | CG12193 | 49835 | GD |
|  |  | Or46a | CG17849 | 4848 | GD |
|  |  | Or67b | CG14176 | 48511 | GD |
|  |  | Or33b | CG16961 | 7583 | GD |
|  |  | Or92a | CG17916 | 36637 | GD |
|  |  | Or47b | CG13206 | 9354 | GD |
|  |  | Or49a | CG13158 | 24498 | GD |
|  |  | Or83c | CG15581 | 42493 | GD |
|  |  | Or67c | CG14156 | 42710 | GD |
|  |  | Or10a | CG17867 | 107609 | KK |
|  |  | Or13a | CG12697 | 104954 | KK |
|  |  | Or19a | CG18859 | 109388 | KK |
|  |  | Or19b | CG32825 | 109050 | KK |
|  |  | Or22b | CG4231 | 105600 | KK |
|  |  | Or23a | CG9880 | 100866 | KK |
|  |  | Or2a | CG3206 | 101021 | KK |
|  |  | Or33a | CG16960 | 101137 | KK |
|  |  | Or33c | CG5006 | 104198 | KK |
|  |  | Or42a | CG17250 | 100316 | KK |
|  |  | Or43a | CG1854 | 110586 | KK |
|  |  | Or43b | CG17853 | 109777 | KK |
|  |  | Or45a | CG1978 | 100201 | KK |
|  |  | Or47a | CG13225 | 100662 | KK |
|  |  | Or49b | CG17584 | 101293 | KK |
|  |  | Or56a | CG12501 | 107231 | KK |
|  |  | Or59b | CG3569 | 107460 | KK |
|  |  | Or59c | CG17226 | 101681 | KK |
|  |  | Or65a | CG32401 | 101699 | KK |
|  |  | Or65b | CG32402 | 103959 | KK |
|  |  | Or65c | CG32403 | 106432 | KK |
|  |  | Or67a | CG12526 | 100323 | KK |
|  |  | Or69a | CG33264 | 102981 | KK |
|  |  | Or71a | CG17871 | 110585 | KK |
|  |  | Or7a | CG10759 | 107874 | KK |
|  |  | Or82a | CG31519 | 104532 | KK |
|  |  | Or85b | CG11735 | 106732 | KK |
|  |  | Or85d | CG11742 | 102428 | KK |
|  |  | Or85e | CG9700 | 101916 | KK |
|  |  | Or85f | CG16755 | 108238 | KK |
|  |  | Or88a | CG14360 | 103815 | KK |
|  |  | Or98a | CG5540 | 104155 | KK |
|  |  | Or98b | CG1867 | 101693 | KK |
|  |  | Or9a | CG15302 | 108062 | KK |
|  |  |  |  |  |  |
|  |  | w^1118^ | GD Control | 60000 |  |
|  |  | y w ;P(attP,y[+],w[3`]) | KK Control | 601000 |  |

**Supplementary Table 2. Hedge’s *g* effect sizes for RNAi and tetanus toxin (-TNTG) lines.** Dark gray rows indicate RNAi lines for specific olfactory receptors (OR) deemed to have unacceptably large background effects (*g* > 0.50 at [0] QMP) and were not included in the response-to-pheromone screen. Light gray rows had relatively minor background effects and were therefore included in the pheromone screen. These lines did not, however, show a significant knockdown effect − that is, they continued to respond to pheromone treatment (*g* > 0.50 at [20] QMP). Finally, white rows represent candidate receptor genes of interest. The RNAi knockdown or -TNTG disruption effectively blocks the worker-like response to queen pheromone (*g* < 0.50 at [20] QMP).

|  |  |  |  |  |  |  |
| --- | --- | --- | --- | --- | --- | --- |
| *Hedge’s g* for ovary egg number | | |  | *Hedge’s g* for ovary area | | |
|  | QMP concentration | |  |  | QMP concentration | |
| OR | [0] | [20] |  | OR | [0] | [20] |
| Or65a | 1.62 | 0.01 |  | Or85e | 1.24 | 0.61 |
| Or19b | 1.37 | 0.26 |  | Or65a | 1.14 | 0.14 |
| Or2a | 0.88 | 1.52 |  | Or19b | 0.95 | 0.17 |
| Or65c | 0.78 | 0.16 |  | Or69a | 0.91 | 0.44 |
| Or85e | 0.78 | 1.1 |  | Or88a | 0.87 | 1.06 |
| Or85d | 0.65 | 1.04 |  | Or2a | 0.83 | 0.86 |
| Or42a | 0.62 | 0.11 |  | Or85f | 0.83 | 0.72 |
| Or67c | 0.6 | 1.02 |  | Or65c | 0.82 | 0.09 |
| Or88a | 0.59 | 0.87 |  | Or67c | 0.75 | 0.62 |
| Or85f | 0.53 | 0.69 |  | Or42a | 0.7 | 0.24 |
| Or67a | 0.53 | 0.83 |  | Or67a | 0.69 | 0.84 |
| Or85a | 0.35 | 1.4 |  | Or85d | 0.66 | 0.83 |
| Or19a | 0.49 | 1.35 |  | Or98b | 0.65 | 0.86 |
| Or33c | 0.44 | 1.25 |  | Or83c | 0.63 | 0.45 |
| Or22b | 0.36 | 1.21 |  | Or45a | 0.62 | 0.49 |
| Or71a | 0.45 | 1.09 |  | Or82a | 0.6 | 0.44 |
| Or33b | 0.07 | 1 |  | Or13a | 0.57 | 0.92 |
| Or98b | 0.45 | 0.98 |  | Or10a | 0.54 | 0.7 |
| Or22a | 0.18 | 0.87 |  | Or19a | 0.53 | 1.38 |
| Or10a | 0.28 | 0.85 |  | Or22b | 0.26 | 1.34 |
| Or13a | 0.21 | 0.85 |  | Or59b | 0.2 | 0.79 |
| Or7a | 0.24 | 0.83 |  | Or71a | 0.45 | 0.77 |
| Or45b | 0.37 | 0.83 |  | Or92a | 0.39 | 0.67 |
| Or92a | 0.2 | 0.8 |  | Or33b | 0.27 | 0.64 |
| Or83c | 0.45 | 0.8 |  | Or49a | 0.4 | 0.61 |
| Or69a | 0.41 | 0.68 |  | Or33c | 0.22 | 0.6 |
| Or49a | 0.38 | 0.67 |  | Or45b | 0.22 | 0.56 |
| Or45a | 0.45 | 0.67 |  | Or67b | 0.09 | 0.54 |
| Or46a | 0.01 | 0.67 |  | Or85a | 0.38 | 0.51 |
| Or67b | 0.09 | 0.67 |  | Or59c | 0.4 | 0.49 |
| Or43b | 0.34 | 0.64 |  | Or47b | 0.14 | 0.47 |
| Or82a | 0.16 | 0.62 |  | Or7a | 0.38 | 0.46 |
| Or59b | 0.1 | 0.59 |  | Or43b | 0.46 | 0.45 |
| Or49b-TNTG | 0.06 | 0.59 |  | Or46a | 0.17 | 0.39 |
| Or59c | 0.06 | 0.53 |  | Or85b | 0.37 | 0.35 |
| Or47b | 0.12 | 0.49 |  | Or43a | 0.06 | 0.34 |
| Or23a | 0.29 | 0.49 |  | Or22a | 0.02 | 0.31 |
| Or33a | 0.03 | 0.49 |  | Or47a | 0.42 | 0.27 |
| Or56a-TNTG | 0.03 | 0.47 |  | Or23a | 0.33 | 0.27 |
| Or85b | 0.14 | 0.42 |  | Or9a | 0.37 | 0.25 |
| Or98a-TNTG | 0.04 | 0.39 |  | Or49b-TNTG | 0.10 | 0.24 |
| Or43a | 0.06 | 0.36 |  | Or56a-TNTG | 0.03 | 0.23 |
| Or47a | 0.12 | 0.36 |  | Or65b | 0.42 | 0.21 |
| Or9a | 0.04 | 0.27 |  | Or98a-TNTG | 0.12 | 0.20 |
| Or65b | 0.15 | 0.2 |  | Or56a | 0.23 | 0.15 |
| Or98a | 0.08 | 0.2 |  | Or98a | 0.06 | 0.08 |
| Or56a | 0.15 | 0.19 |  | Or33a | 0.08 | 0.05 |
| Or49b | 0.44 | 0.13 |  | Or49b | 0.4 | 0.02 |

**Supplementary Table 3. Substructure similarity scores between each receptor's predicted ligand and the five (effectively four) components of QMP. Light grey shading shows the single highest score between ethyl benzoate (*Or98a*) and HOB, while dark grey shading shows the single highest scores for the other components of QMP.**

| Receptor | Predicted ligand | 9-ODA | *cis-*/*trans-* | HOB | HVA |
| --- | --- | --- | --- | --- | --- |
|  |  |  | 9-HDA |  |  |
| Or7a | E2-hexenal | 0.5384 | 0.5 | 0.3846 | 0.4615 |
| Or9a | 3-hydroxy-2-butanone | 0.3571 | 0.333 | 0.3077 | 0.2857 |
| Or22a | methyl octanoate | 0.5 | 0.4706 | 0.375 | 0.2778 |
| Or22a | ethyl hexanoate | 0.4375 | 0.4118 | 0.4 | 0.2941 |
| Or22a | isobutyl acetate | 0.2353 | 0.2222 | 0.3571 | 0.25 |
| Or23a | 1-pentanol | 0.3571 | 0.4286 | 0.3077 | 0.3846 |
| Or33a | 2-heptanone | 0.6154 | 0.4667 | 0.2667 | 0.25 |
| Or43a | 1-hexanol | 0.4285 | 0.5 | 0.2857 | 0.3571 |
| Or43a | cyclohexanol | 0.333 | 0.4 | 0.2857 | 0.3571 |
| Or43b | ethyl trans-2-butenoate | 0.4 | 0.375 | 0.5833 | 0.4286 |
| Or43b | ethyl butyrate | 0.3125 | 0.2941 | 0.4615 | 0.333 |
| Or46a | 4-methylphenol | 0.4 | 0.375 | 0.7273 | 0.6667 |
| Or47a | butyl acetate | 0.2353 | 0.2941 | 0.3571 | 0.333 |
| Or47a | pentyl acetate | 0.2941 | 0.3529 | 0.333 | 0.3125 |
| Or47b | gama butyrolactone | 0.3571 | 0.25 | 0.4167 | 0.2857 |
| Or49b | 2-methylphenol | 0.3125 | 0.2941 | 0.5833 | 0.5385 |
| Or56a | geosmin | 0.4211 | 0.4737 | 0.1905 | 0.2381 |
| Or59c | 3-octanol | 0.5714 | 0.5333 | 0.25 | 0.3125 |
| Or85b | e2-hexenol | 0.5385 | 0.5 | 0.5 | 0.5833 |
| Or85b | 6-methyl-5-hepten-2-one | 0.4667 | 0.4375 | 0.333 | 0.4 |
| Or98a | ethyl benzoate | 0.333 | 0.3158 | 0.8333 | 0.4375 |
